# Supplementary material for: Molecular models of multiple sclerosis severity identify heterogeneity of pathogenic mechanisms
Source: Nat Commun. 2022 Dec 12;13:7670. doi: 10.1038/s41467-022-35357-4 (PMC9744737; doi:10.1038/s41467-022-35357-4)
Supplement: Supplementary file 1 — Supplementary Information [file 41467_2022_35357_MOESM1_ESM.pdf]

Figure S1

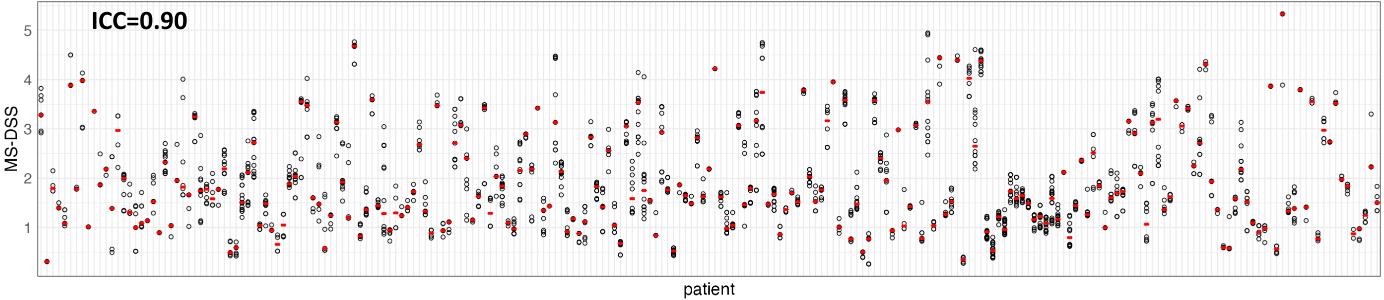

Figure S1: Inter-class correlation coefficient (ICC) for MS-DSS outcomes measured in the longitudinal cohort of MS patients ($N_{patients}$=227, N_{visits}=1538). Empty black circles represent measured MS-DSS, red bars represent patient's median.

Figure S2

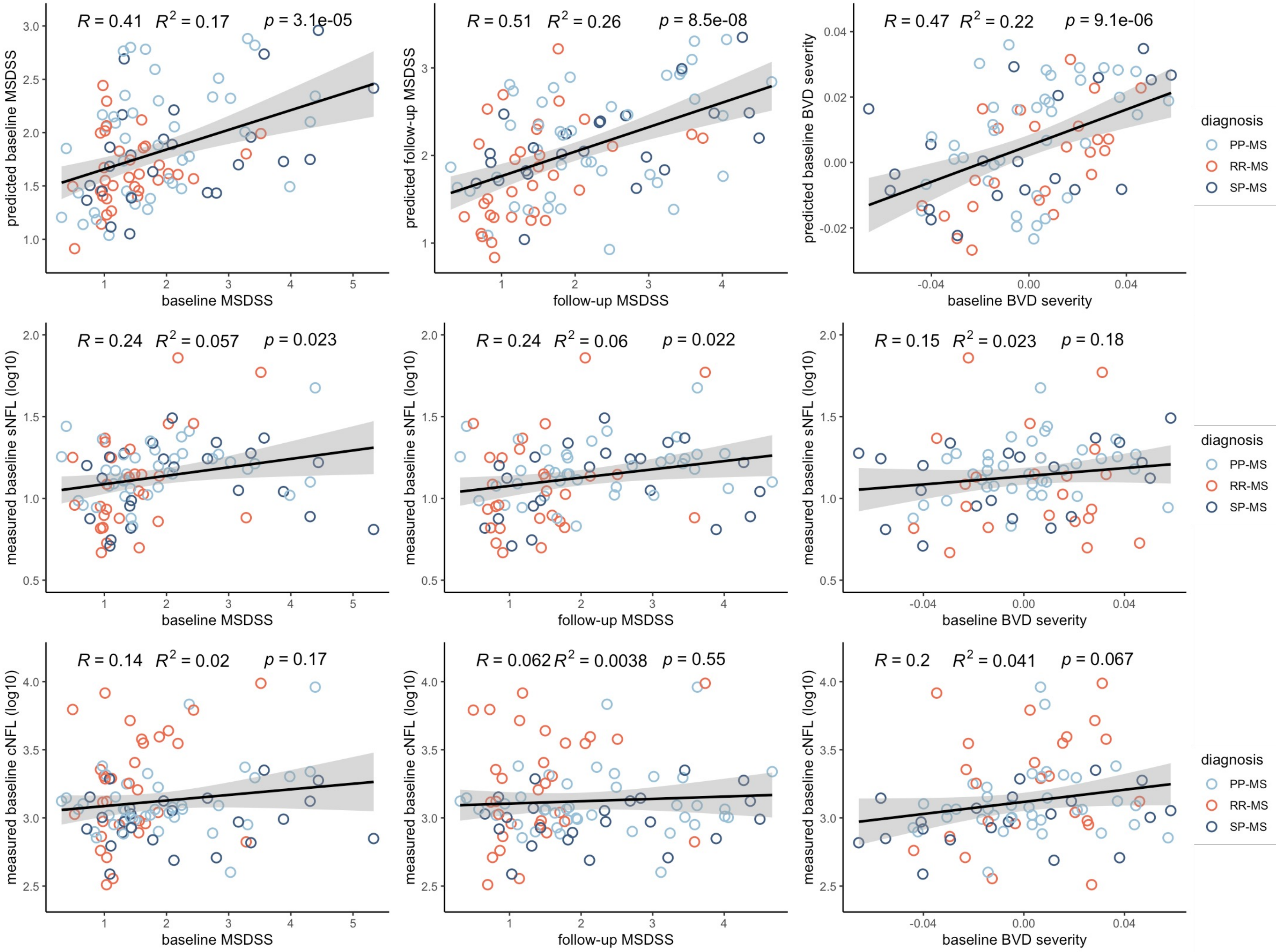

Figure S2: Correlation between measured MS severity outcomes (x-axes) and CSF biomarker-predicted MS severity outcomes (y-axes top row plots), measured baseline serum NFL (y-axes, middle row plots), and measured baseline CSF NFL (y-axes, bottom row plots) in the validation cohort. R was calculated using Pearson correlation test, Coefficient of determination (R2) and corresponding p-value were generated from a linear regression model between observed and predicted values. Linear regression lines are shown in black with gray-shaded error band representing 95% confidence interval. All statistical tests were two-sided.
