# Supplementary material for: Molecular models of multiple sclerosis severity identify heterogeneity of pathogenic mechanisms
Source: Nat Commun. 2022 Dec 12;13:7670. doi: 10.1038/s41467-022-35357-4 (PMC9744737; doi:10.1038/s41467-022-35357-4)
Supplement: Supplementary file 3 — Description of Additional Supplementary Files [file 41467_2022_35357_MOESM3_ESM.pdf]

Description of Additional Supplementary Files

Supplementary Data 1: Search Tool for the Retrieval of Interacting Genes/Proteins (STRING) annotation for age associated SOMAmers with concordant directionality between healthy volunteers and multiple sclerosis patients. See also Figure 2.

Supplementary Data 2: Search Tool for the Retrieval of Interacting Genes/Proteins (STRING) annotation for age associated SOMAmers with discordant directionality between healthy volunteers and multiple sclerosis patients. See also Figure 2.

Supplementary Data 3: Search Tool for the Retrieval of Interacting Genes/Proteins (STRING) annotation for SOMAmers that are elevated in females. See also Figure 3.

Supplementary Data 4: Search Tool for the Retrieval of Interacting Genes/Proteins (STRING) annotation for SOMAmers that are elevated in males. See also Figure 3.

Supplementary Data 5: Functional enrichment analyses of SOMAScan proteins measured in CSF samples of MS patients. See also Figure 5.

Supplementary Data 6: Spearman correlations (two-sided) and False Discovery Rate adjusted p-values between all SOMAmers and various multiple sclerosis severity measures in untreated patients.

Supplementary Data 7: Annotated SOMAmer ratios for three statistical learning models of multiple sclerosis severity. Spearman correlations (two-sided) and False Discovery Rate adjusted p-values are showen. See also Figure 6 and 8.

Supplementary Data 8: Correlation matrix of measured and predicted MS severity outcomes generated using Spearman correlation test (two-sided) and False Discovery Rate adjusted p-values.

Supplementary Data 9: Search Tool for the Retrieval of Interacting Genes/Proteins (STRING) annotation for SOMAmers present in the myeloid lineage/TNF module or “module 1”. See also Figure 8.

Supplementary Data 10: Search Tool for the Retrieval of Interacting Genes/Proteins (STRING) annotation for SOMAmers present in the central nervous system (CNS) repair module or “module 2”. See also Figure 8.

Supplementary Data 11: Search Tool for the Retrieval of Interacting Genes/Proteins (STRING) annotation for SOMAmers present in the complement/coagulation module or “module 3”. See also Figure 8.

Supplementary Data 12: Search Tool for the Retrieval of Interacting Genes/Proteins (STRING) annotation for SOMAmers present in the adaptive immunity and central nervous system (CNS) stress module or “module 4”. See also Figure 8.

Supplementary Data 13: Raw data.

Supplementary Data 14: Input data and R codes. The R code generates models predicting MS-DSS at baseline, MS-DSS at follow up visit, and Brain volume decrease (BVD) severity. The code generates ratios of Somamers selected for each model, performs sex/gender adjustment based on Healthy Volunteer dataset and builds Random Forest models. These models then predict the three outcomes. The "input" folder contains all files necessary to run the code, the predictions and models are saved in the "output" folder.

Supplementary Data 15: List of genes associated with age displayed in heatmap in Figure 2C.

Supplementary Data 16: List of genes comprising 4 modules in Figure 8a-b.
