# Supplementary material for: Molecular models of multiple sclerosis severity identify heterogeneity of pathogenic mechanisms
Source: Nat Commun. 2022 Dec 12;13:7670. doi: 10.1038/s41467-022-35357-4 (PMC9744737; doi:10.1038/s41467-022-35357-4)
Supplement: Supplementary file 20 — Reporting Summary [file 41467_2022_35357_MOESM20_ESM.pdf]

nature portfolio

Corresponding author(s): Bibiana Bielekova

Last updated by author(s): Nov 9, 2022

Reporting Summary

Nature Portfolio wishes to improve the reproducibility of the work that we publish. This form provides structure for consistency and transparency in reporting. For further information on Nature Portfolio policies, see our Editorial Policies and the Editorial Policy Checklist.

Statistics

For all statistical analyses, confirm that the following items are present in the figure legend, table legend, main text, or Methods section.

n/a	Confirmed	
☐	☒	The exact sample size (*n*) for each experimental group/condition, given as a discrete number and unit of measurement
☐	☒	A statement on whether measurements were taken from distinct samples or whether the same sample was measured repeatedly
☐	☒	The statistical test(s) used AND whether they are one- or two-sided<br>*Only common tests should be described solely by name; describe more complex techniques in the Methods section.*
☐	☒	A description of all covariates tested
☐	☒	A description of any assumptions or corrections, such as tests of normality and adjustment for multiple comparisons
☐	☒	A full description of the statistical parameters including central tendency (e.g. means) or other basic estimates (e.g. regression coefficient) AND variation (e.g. standard deviation) or associated estimates of uncertainty (e.g. confidence intervals)
☐	☒	For null hypothesis testing, the test statistic (e.g. *F*, *t*, *r*) with confidence intervals, effect sizes, degrees of freedom and *P* value noted<br>*Give P values as exact values whenever suitable.*
☒	☐	For Bayesian analysis, information on the choice of priors and Markov chain Monte Carlo settings
☒	☐	For hierarchical and complex designs, identification of the appropriate level for tests and full reporting of outcomes
☒	☐	Estimates of effect sizes (e.g. Cohen's *d*, Pearson's *r*), indicating how they were calculated

Our web collection on statistics for biologists contains articles on many of the points above.

Software and code

Policy information about availability of computer code

Data collection	Clinical data was collected by board-certified neurologists and non-medical staff and recorded in our research database with integrated NeurEx(TM) App (originally published here: https://doi.org/10.1002/acn3.640) that automatically generates traditional MS disability scores (e.g., EDSS, SNRS). CombiWISE scores were calculated using published formula (https://doi.org/10.3389/fneur.2016.00131). MS-DSS scores were generated using previously published model (https://doi.org/10.3389/fneur.2017.00598). Volumetric MRI data were generated using published Lesion-TOADS algorithm (https://doi.org/10.1016/j.neuroimage.2009.09.005) implemented into QMENTA platform (https://support.qmenta.com/knowledge/lesion-toads-workflow).
Data analysis	Data analysis was performed using R Studio software Version 1.1.463 utilizing R Version 3.6.1 running custom codes available in the Supplementary Information. Random Forest pipeline for model generation was run on NIH High Performing Computation Biowulf Cluster. Biological interpretation of selected CSF biomarkers was assessed using STRING v10 web resource (https://string-db.org/) g:Profiler web resource (https://biit.cs.ut.ee/gprofiler/gost).

For manuscripts utilizing custom algorithms or software that are central to the research but not yet described in published literature, software must be made available to editors and reviewers. We strongly encourage code deposition in a community repository (e.g. GitHub). See the Nature Portfolio guidelines for submitting code & software for further information.

Data

Policy information about availability of data

All manuscripts must include a data availability statement. This statement should provide the following information, where applicable:

- Accession codes, unique identifiers, or web links for publicly available datasets
- A description of any restrictions on data availability
- For clinical datasets or third party data, please ensure that the statement adheres to our policy

All relevant raw data supporting key findings of this study are available within this article and its Supplementary Information.

Human research participants

Policy information about studies involving human research participants and Sex and Gender in Research.

Reporting on sex and gender	We report sex in our study and we performed sex-adjustment of somamer levels, reflecting the biology associated with sexual dimorphism.
Population characteristics	Two population of subjects were were prospectively recruited:<br>1. Subjects with a definitive diagnosis of Multiple Sclerosis that were not in exacerbation or on low-efficacy disease modifying therapy within 3 months of lumbar puncture, or on high-efficacy disease modifying therapy within 6 months of lumbar puncture. The average age of this population was 49.7 (SD +/- 11.7) years, represented by 54% to 46% females to males distribution.<br>2. Healthy volunteers that lack neurological neurological diagnosis or systemic disease that could influence neurological disability or brain MRI, and with vital signs in the normal range during the initial screening. The average age of this population was 41.6 (SD 11.7) years, with 52% to 48% females to males distribution.
Recruitment	Subjects were recruited prospectively as part of the Natural History protocol “Comprehensive Multimodal Analysis of Neuroimmunological Diseases of the Central Nervous System” (Clinicaltrials.gov identifier NCT00794352)
Ethics oversight	The study was reviewed and approved by the Intramural Institutional Review Board at the National Institutes of Health.

Note that full information on the approval of the study protocol must also be provided in the manuscript.

Field-specific reporting

Please select the one below that is the best fit for your research. If you are not sure, read the appropriate sections before making your selection.

☒ Life sciences ☐ Behavioural & social sciences ☐ Ecological, evolutionary & environmental sciences

For a reference copy of the document with all sections, see nature.com/documents/nr-reporting-summary-flat.pdf

Life sciences study design

All studies must disclose on these points even when the disclosure is negative.

Sample size	No power calculation was performed, all available samples were randomly split into training and validation cohort 2:1, upon revision, we have added additional samples into the validation cohort, observing minimal effect on the model performance in the validation cohort (achieving low p-value in the original, as well as the extended validation cohort).
Data exclusions	No data were excluded
Replication	Single set of clinical, volumetric MRI, and CSF biomarker values were collected for each patient/visit. No replication was attempted for these outcomes. However, the final CSF biomarker-based models generated in the training dataset were validated in the independent validation cohort that did not contribute in any way to the development of the models. Moreover, addition of more samples into the validation cohort didn't significantly effect the model performance.
Randomization	The blinded data were randomly split into training and validation cohort (2:1) before any analyses were performed.
Blinding	Clinical, imaging and demographic data were collected prospectively before the proteomic Somascan assay was performed on CSF samples. The data were QC-ed and locked in the research database. Somascan assay was performed on coded samples by personnel blinded to any metadata associated with the samples.

Reporting for specific materials, systems and methods

We require information from authors about some types of materials, experimental systems and methods used in many studies. Here, indicate whether each material, system or method listed is relevant to your study. If you are not sure if a list item applies to your research, read the appropriate section before selecting a response.

Materials & experimental systems

n/a	Involved in the study
☒	☐ Antibodies
☒	☐ Eukaryotic cell lines
☒	☐ Palaeontology and archaeology
☒	☐ Animals and other organisms
☐	☒ Clinical data
☒	☐ Dual use research of concern

Methods

n/a	Involved in the study
☒	☐ ChIP-seq
☒	☐ Flow cytometry
☐	☒ MRI-based neuroimaging

Clinical data

Policy information about clinical studies

All manuscripts should comply with the ICMJE guidelines for publication of clinical research and a completed CONSORT checklist must be included with all submissions.

Clinical trial registration	NCT00794352
Study protocol	Study protocol was downloaded from IRB website and is made available to peer reviewers.
Data collection	Data were prospectively collected at the NIH between May 2004 - April 2021
Outcomes	The primary and secondary outcomes are defined in the Natural History study protocol. The primary outcome is a definitive diagnosis of MS or other disorder. Secondary outcomes relevant to the manuscript are clinical disability measured by NeurEx and CombiWISE (see the protocol for details). MS-DSS as an outcome used for development of the models presented in the study was described here: Weideman AM, et al. New Multiple Sclerosis Disease Severity Scale Predicts Future Accumulation of Disability. Front Neurol 8, 598 (2017). The MRI severity outcome was based on MRI volumetric data generated by Qmenta platform (https://www.qmenta.com) from structural images obtained at NIH.

Magnetic resonance imaging

Experimental design

Design type	We performed one set of structural MRI scans (T1-weighted, T2-weighted images) at each clinic visit for each subject.
Design specifications	NA
Behavioral performance measures	NA

Acquisition

Imaging type(s)	structural
Field strength	1.5T and 3T
Sequence & imaging parameters	T1 magnetization-prepared rapid gradient-echo (MPRAGE) or fast spoiled gradient-echo (FSPGR) and T2 weighted three-dimensional fluid attenuation inversion recovery (3D FLAIR).
Area of acquisition	whole brain

Diffusion MRI ☐ Used ☐ Not used

Preprocessing

Preprocessing software	Raw unprocessed but locally anonymized and encrypted T1 - MPRAGE or T1 - FSPGR and T2 - 3D FLAIR DICOM files as input sequences, ideally with 1 mm3 isotropic resolution, were uploaded to the QMENTA platform. LesionTOADS, now implemented into the cloud-based service, is a fully automated segmentation algorithm using multichannel MRI data. The uploaded sequences are anterior commissure-posterior commissure (ACPC) aligned, rigidly registered to each other and skull stripped (the T1 image is additionally bias-field corrected). The segmentation is performed by using an atlas-based technique combining a topological and statistical atlas resulting in computed volumes for each segmented tissue in mm3.
Normalization	NA
Normalization template	Data were not normalized
Noise and artifact removal	NA

Volume censoring | NA

Statistical modeling & inference

Model type and settings | NA

Effect(s) tested | NA

Specify type of analysis: ☐ Whole brain ☐ ROI-based ☐ Both

Statistic type for inference (See Eklund et al. 2016) | NA

Correction | NA

Models & analysis

n/a	Involved in the study
☒	☐ Functional and/or effective connectivity
☒	☐ Graph analysis
☐	☒ Multivariate modeling or predictive analysis

Multivariate modeling and predictive analysis | Random forest algorithm was used to construct the CSF biomarker-based models of MS severity. All possible protein ratios were included in the modeling along with individual markers as independent variables. Prior to model development, the available data were randomly split into training and validation cohorts, with 2/3 being used as a training cohort and 1/3 retained for model validation. The number of ratios/markers was reduced based on predictive performance. 10 random forests were run using the training cohort, and variable importance measures based on node impurity were averaged together. The bottom 10% of variables, according to these average variable importance measures, were removed from the candidate set. This process was repeated until only three variables remained. The mean and standard deviation of the out-of-bag error was graphically assessed to determine the final cut point for each model. For each instance, a final random forest model was constructed in the training cohort and it's performance was tested in the independent validation cohort.
